# Role of calcification in J-CTO score: a viewpoint of intraplaque guidewire tracking techniques

**DOI:** 10.1080/07853890.2024.2396076

**Published:** 2024-08-28

**Authors:** Shih-Chi Liu, Chien-Lin Lee, Jen-Fang Cheng, Jiunn-Yang Chiang, Cheng-Ting Tsai, Chi-Jen Chang, Chia-Pin Lin, Chi-Hung Huang, Jun-Ting Liou, Chia-Ti Tsai, Yi-Chih Wang, Juey-Jen Hwang

**Affiliations:** a Cardiovascular Division, Department of Internal Medicine, Fu Jen Catholic University Hospital, New Taipei City, Taiwan; b Cardiovascular Division, Department of Internal Medicine, Far Eastern Memorial Hospital, New Taipei City, Taiwan; c Cardiovascular Division, Department of Internal Medicine, National Taiwan University Hospital, Taipei, Taiwan; d Cardiovascular Division, Department of Internal Medicine, MacKay Memorial Hospital, Taipei, Taiwan; e Cardiovascular Division, Department of Internal Medicine, Chang Gung Memorial Hospital, Taoyuan, Taiwan; f Cardiovascular Division, Department of Internal Medicine, Cathay General Hospital, Taipei, Taiwan; g Cardiovascular Division, Department of Internal Medicine, China Medical University Hsinchu Hospital, Zhubei City, Taiwan

**Keywords:** chronic total occlusion, calcium, intraplaque tracking, intervention, J-CTO score

## Abstract

**Background:**

As the burden and distribution of calcification within chronic total occlusion (CTO) lesions can be diverse, its effect on CTO recanalization using multiple devices and techniques is debatable. This study investigated the role of calcification in wiring-based intraplaque tracking techniques for CTO recanalization.

**Methods:**

A modified J-CTO score without counting calcification was used to analyze the procedures of 458 consecutive patients who underwent CTO interventions. Failed guidewire crossing and intraplaque tracking were considered procedural failures. Recanalization time details were analyzed for successful procedures.

**Results:**

In patients with calcified CTO, the rate of procedural success only significantly declined to be lower than that of noncalcified CTO when the modified J-CTO score was ≥3 (77% vs. 94%, *p* = 0.008). In 422 patients with successful procedures, the presence of calcification was irrelevant to guidewire crossing time, but was accompanied with longer time from guidewire cross to final angiogram when the modified J-CTO score was 1–2 (53 ± 35 vs. 35 ± 17 [noncalcified] min, *p* < 0.001). Multivariate analyses showed that calcification was independently associated with procedural failure (odds ratio [OR] = 5.1, 95% confidence interval [CI] = 1.4–18.3) in lesions with modified J-CTO ≥3, and prolonged angioplasty/stenting procedures >60 min (OR = 4.8, 95% CI = 2.2–10.2) in successfully recanalized lesions with modified J-CTO score 1–2.

**Conclusions:**

Using intraplaque guidewire tracking, calcification was unfavorable for very difficult CTO lesions, and caused prolongation of angioplasty time for lesions with moderate complexity. This suggested that the role of calcification in the J-CTO score could be altered when different recanalization techniques were applied for CTO interventions.

## KEY MESSAGES

Since several commonly used scoring systems for grading the difficulty of CTO-PCI are derived from multiple recanalization techniques and devices, their application should be fundamental. However, most CTO interventionists usually have their own favored recanalization techniques in the real-world. As one of the parameters of J-CTO score, the findings of the study suggest that the interpretation of calcification during CTO-PCI could be altered and should be cautious if different recanalization technique was used.

## Introduction

Chronic total occlusion (CTO) is a challenging coronary lesion subset and accounts for approximately 15% of all coronary interventions [1, 2]. As the occluded duration is longer, the composition within CTO typically becomes dense with more heavily calcific deposits [3, 4]. In real-world data, the incidence rate of moderate or severe calcium within CTO segments is 57–59% [5–7]. Apart from the Japan CTO (Multicenter Chronic Total Occlusion Registry of Japan: J-CTO) score [8], several scoring systems of CTO-PCI [9–11] have shown that the presence of calcification can serve as a fundamental parameter for grading the difficulty of recanalization. However, these scoring systems include multiple recanalization devices and techniques that may partly jeopardize the true contribution of calcification in the evaluation of the overall technical difficulty. For example, evident calcification is associated with more application of “dissection and reentry” and retrograde techniques [5]. Theoretically, the difficulty and procedure time required for recanalizing CTO lesions with calcification must be not identical between operators favoring the “dissection and reentry” methods earlier in the procedure and those focusing more on the wiring-based “intraplaque tracking” techniques.

Although the application of reentry devices and several specific retrograde methods have been recommended to facilitate the success of CTO-PCI [12–14], their accessibility and the learning curve required remain crucial in the real-world practice. For most interventionists, either antegrade or retrograde wiring-based intraplaque tracking and crossing techniques are still the basic and main strategy. The presence of calcification usually suggests a harder CTO lesion [3], but the distribution of calcification can serve as a landmark to ensure that the guidewire is close to the true lumen through the occluded route during guidewire tracking, particularly when the lesion is remarkably long to determine the exact vessel course. As the role of calcification in CTO-PCI with respect to the techniques used remains debatable, we aimed to test it by assessing the technical feasibility and procedural time of CTO interventions from the viewpoint of intraplaque guidewire tracking techniques.

## Methods

### Study design and population

**Figure 1.**
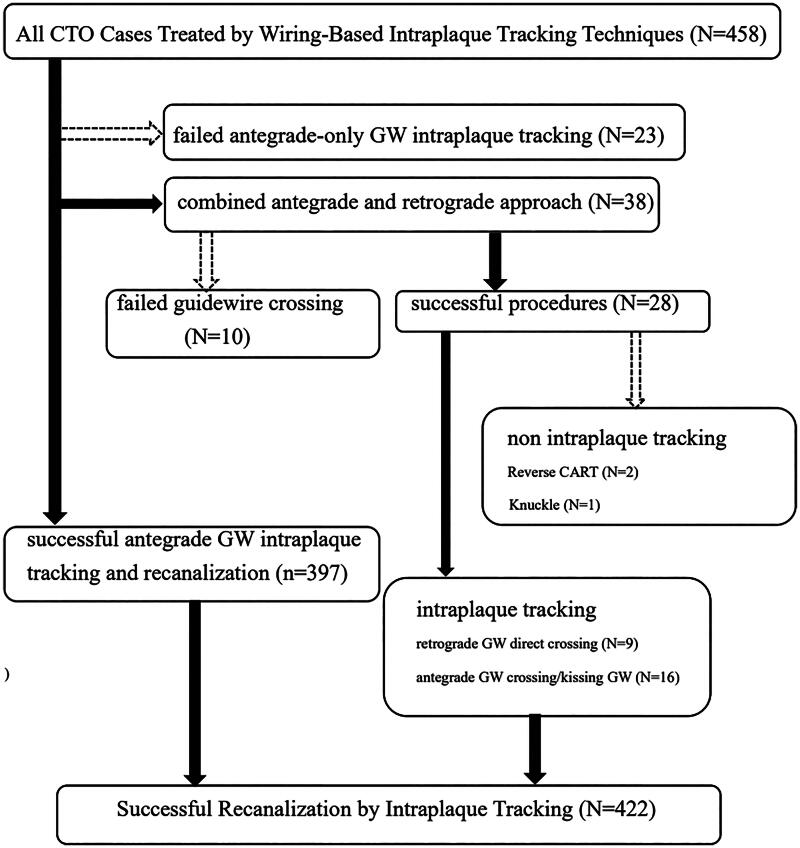
The 458 cases with CTO interventions by wiring-based intraplaque tracking techniques. Successfully wiring was done in 397 of 420 cases with antegrade-only techniques. Thirty-eight cases required the back-up of retrograde techniques, and 28 cases had successful recanalization. The two cases with reverse CART and one with knuckle wire techniques were considered as non-intraplaque tracking. An overall of 422 cases with successful true-lumen tracking were included for analyzing the procedure time. CTO, chronic total occlusion; CART, controlled antegrade and retrograde tracking; GW, guidewire.

This prospective registry study was initiated in 2019 and approved by the Ethics Committee of the National Taiwan University Hospital (201904023RINC). In addition, retrospective data collection from August 2014 was allowed and was also approved by the ethics committee (201907064RIND). The period of retrospective data collection was determined by the introduction of Gaia series (Asahi Intecc Medical, Japan) guidewires in our hospital, because the Gaia series guidewire was particularly designed for intraplaque tracking which was very essential to facilitate either antegrade or retrograde wiring techniques we used in the study. Written informed consent was obtained from the participants. In total, 458 consecutive patients (64 ± 11 years old, 403 men) who underwent CTO-PCI using intraplaque tracking techniques between August 2014 and March 2022 were included in the analyses (Figure 1). Wiring-based intraplaque tracking was conceptualized by Taiwan True-Lumen Tracking club initiated by seven high-volume CTO operators from independent medical centers in north Taiwan, and the purpose was to minimize the extent of subintima creation and stenting, but not intend to ensure a complete intraplaque course. Additionally, successful “intraplaque crossing” was defined as no loss of antegrade flow for any side branch ≥1.5 mm from 5 mm before to 5 mm after the occluded segment in final coronary angiography [15]. Based on the study design to clarify the role of calcification, a modified J-CTO score excluding the count of calcification was used for representation of lesion complexity on top of the presence or absence of calcification. Patients with chronic kidney disease (CKD) had an estimated glomerular filtration rate <60 ml/min/1.73 m^2^. Patients with congestive heart failure had heart failure signs/symptoms meeting the Framingham criteria and left ventricular ejection fraction <50% before the index procedure.

### Procedures of intraplaque guidewire tracking

A CTO lesion was confirmed if the flow was thrombolysis in myocardial infarction (MI) grade 0 with the duration for at least 3 months [16, 17]. Multivessel disease was defined as the presence of stenoses ≥50% in at least two major epicardial coronary arteries by quantitative coronary angiography. Evident target lesion calcification was described as radiopacities observed during the whole cardiac cycle or without cardiac motion before contrast injection [18]. The judgment of calcification and the other parameters of modified J-CTO score was assessed by two independent operators (Dr. SC Liu and Dr. CL Lee).

Even in the presence of ambiguous proximal cap, poor distal vessel quality, or bifurcation at distal cap, we still tried antegrade wiring first to overcome each unfavorable anatomy by guidewire manipulation, escalation, and parallel wire technique [15]. The timing of switching to a parallel-wire or retrograde approach was determined by the interventionist. When retrograde procedures were required, guidewires with softer tip load (mainly ≤1 g) for direct crossing or serving as the landmark for antegrade guidewire kissing were the preferred techniques. Reverse controlled antegrade and retrograde tracking (CART) or knuckle wire techniques were used only for rescue. However, neither technique, even with successful recanalization, was not considered successful intraplaque tracking procedures to minimize the possible confounding to the study purpose. The total number of guidewires with different tip load used for recanalization was counted. For example, the tip load of XTR, XT, and XTA (Asahi Intecc Medical, Japan) is 0.6, 0.8, and 1.0 gram, respectively. We considered them as three different tip loads in the study. If any of the sizable side branches ≥1.5 mm was lost after balloon dilatation or stenting even with intraplaque crossing, we always tried to restore the antegrade flow during the angioplasty procedures to fulfil our principle. In the study, “dissection and reentry” devices were never used, and intravascular imaging was recommended but not mandatory. In addition to the criteria for intraplaque tracking/crossing, restoration of thrombolysis in MI grade 3 anterograde flow, postprocedural stenosis of <30%, and no occurrence of in-hospital major adverse cardiac and/or cerebrovascular events, including cardiac death, Q-wave MI, stroke, or any repeat target lesion revascularization, were also necessary for the definition of procedural success. In patients with procedural success, the times for guidewire crossing, guidewire crossing to the final angiogram, and the total procedure were recorded.

### Statistical analyses

In statistical testing, two-sided p value ≤0.05 was considered statistically significant. The distributional properties of continuous variables are expressed as means ± standard deviations, and categorical variables are expressed as frequencies and percentages. In the univariate analysis, the unadjusted effect of each parameter was examined using the Wilcoxon rank-sum, chi-squared, or Fisher’s exact test, as appropriate for the data type. Among the 422 patients with successful intraplaque tracking and recanalization, comparisons of the time for guidewire crossing, guidewire crossing to final angiogram, and total procedure across each modified J-CTO score were performed using one-way analysis of variance (ANOVA). Next, multivariate analysis was performed using logistic regression analysis to calculate odds ratio (OR) and 95% confidence interval (CI) to select a set of independent predictors of technique failure among patients with CTO-PCI and prolonged time of angioplasty >60 min among those with successful intraplaque tracking and recanalization. For multivariate analyses, factors based on p-values <0.10 in univariate analyses and prior knowledge were included. All analyses were performed using the IBM SPSS version 25 software package (IBM Corp.).

## Results

### Comparisons of clinical features

Among all patients with CTO interventions shown in Table 1, those with calcified lesions (*N* = 247) were older (66.5 ± 11.1 vs. 61.9 ± 11.0 years, *p* < 0.001) with lower body mass index (25.8 ± 3.9 vs. 26.5 ± 3.7 kg/m^2^, *p* = 0.036) and higher percentage of diabetes (48 vs. 34%, *p* = 0.002) and CKD (31 vs. 21%, *p* = 0.012) compared with those without calcification (*N* = 211). Other clinical features were similar between the two groups.

**Table 1.** Comparisons of clinical features in patients with intraplaque tracking techniques for CTO lesions with and without evident calcification.

	All (*N* = 458)	Calcium (+) (*N* = 247)	Calcium (-) (*N* = 211)	P-value
Age (yrs)	64.4 ± 11.3	66.5 ± 11.1	61.9 ± 11.0	<.001
Male (%)	403 (88%)	212 (86%)	191 (91%)	.124
BMI (kg/m^2^)	26.1 ± 3.8	25.8 ± 3.9	26.5 ± 3.7	.036
Hypertension (%)	355 (78%)	197 (80%)	158 (75%)	.214
Diabetes (%)	189 (41%)	118 (48%)	71 (34%)	.002
Dyslipidemia (%)	340 (74%)	189 (77%)	151 (72%)	.228
Smoking (%)	163 (36%)	95 (38%)	68 (32%)	.166
Old MI (%)	102 (22%)	56 (23%)	46 (24%)	.824
CABG (%)	35 (8%)	22 (9%)	13 (6%)	.271
CHF (%)	114 (25%)	67 (27%)	47 (22%)	.232
eGFR < 60 ml/min/1.73m^2^ (%)	121 (26%)	77 (31%)	44 (21%)	.012
Stroke (%)	29 (6%)	18 (7%)	11 (5%)	.365

BMI, body mass index; CABG, coronary artery bypass graft; CHF, congestive heart failure; CTO, chronic total occlusion; eGFR, estimated glomerular filtration rate; MI, myocardial infarction.

### Procedural details and comparisons of angiographic and procedural characteristics

In Figure 1, the percentage of retrograde techniques in the cohort was 8%. The average time of switching from antegrade to retrograde approach in the study was 71 ± 32 min. Reverse CART or knuckle wire techniques for rescue were used as a minority of retrograde and overall procedures (8% and 0.7%, respectively). We lost significant side branches which patency were intended to keep in 4 of the 23 patients with failed antegrade-only intraplaque guidewire tracking, and in 1 of the 10 patients with failed wiring even with the aid of retrograde techniques. All these patients with loss of sizable side branches were considered as procedural failure in the study.

There are more calcified lesions than non-calcified lesions in the left anterior descending artery (43 vs. 30%, *p* = 0.004), whereas in the left circumflex artery there are more non-calcified lesions than calcified ones (25 vs. 15%, *p* = 0.009) (Table 2). Most interventions were performed *via* all transradial access, but the percentage was relatively lower in patients with calcified occlusions than those without (66 vs. 81%, *p* < 0.001). With respect to lesion characteristics, the J-CTO score was significantly higher in patients with calcification than in those without calcification (3.0 ± 1.2 vs. 1.6 ± 1.2, *p* < 0.001). Even excluding the parameter of calcification, the modified J-CTO score in patients with calcified CTO remained higher than those without (2.0 ± 1.2 vs. 1.6 ± 1.2, *p* < 0.001) owing to a higher percentage of blunt stump (58 vs. 44%, *p* = 0.002), occlusion ≥2 cm (58 vs. 50%, *p* = 0.068), and bending >45° (64 vs. 50%, *p* = 0.002). Moreover, fewer intrastent occlusions (9 vs. 20%, *p* < 0.001) and more use of rotational atherectomy (6% vs. 0, *p* < 0.001) and antegrade guidewires with ≥3 different tip loads (40 vs. 24%, *p* < 0.001) were noted in patients with calcified CTO compared with those with noncalcified CTO. The success rate of intraplaque guidewire tracking for CTO with calcification was 88%, lower than that for CTO without calcification (97%, *p* = 0.001). The overall procedure time (91 ± 56 vs. 69 ± 49 min, *p* < 0.001) and fluoroscopy time (63 ± 35 vs. 50 ± 32, *p* < 0.001) were both longer in patients with calcified CTO compared with those with noncalcified CTO, but the dosage of radiation was comparable between the two groups. With a similar average stent size, the total length of the stents used for calcified CTO lesions was longer than that for noncalcified lesions (60 ± 28 vs. 50 ± 27 mm, *p* = 0.001).

**Table 2.** Comparisons of angiographic and procedural characteristics in patients with intraplaque tracking techniques for CTO lesions with and without evident calcification.

	All (*N* = 458)	Calcium (+) (*N* = 247)	Calcium (-) (*N* = 211)	P-value
Multivessel dz. (N)	412(90%)	224(91%)	188(89%)	.574
Lesion Location (N)				.
LAD (N)	169(37%)	106(43%)	63(30%)	.004
LCX (N)	89(19%)	37(15%)	52(25%)	.009
RCA (N)	195(43%)	103(42%)	92(44%)	.682
LM (N)	1(0.2%)	1(0.4%)	0	.356
SVG (N)	4(0.9%)	0	4(2%)	.030
All transradial (N)	333(73%)	162(66%)	171(81%)	<.001
All transfemoral (N)	76(17%)	45(18%)	31(15%)	.313
Radial + Femoral (N)	49(11%)	40(16%)	9(4%)	<.001
J-CTO score	2.3 ± 1.4	3.0 ± 1.2	1.6 ± 1.2	<.001
J-CTO score minus calcification	1.8 ± 1.2	2.0 ± 1.2	1.6 ± 1.2	<.001
Blunt stump (N)	235(51%)	143(58%)	92(44%)	.002
Occlusion ≧20mm (N)	249(54%)	144(58%)	105(50%)	.068
Bending >45°(N)	264(58%)	159(64%)	105(50%)	.002
Retry case (N)	63(14%)	38(15%)	25(12%)	.274
Instent restenosis (N)	65(14%)	22(9%)	43(20%)	<.001
Intravascular Imaging (N)	206(45%)	117(47%)	89(42%)	.267
IVUS (N)	194(42%)	112(45%)	82(39%)	.162
OCT (N)	12(3%)	5(2%)	7(3%)	.389
Multivessel PCI (N)	189(41%)	94(38%)	95(45%)	.132
Retrograde approach (N)	38(8%)	22(9%)	16(8%)	.610
Parallel wire (N)	53(12%)	33(13%)	20(9%)	.191
Antegrade GW≧3 (N)	151(33%)	100(40%)	51(24%)	<.001
RA (N)	15(3%)	15(6%)	0	<.001
Contrast volume (ml)	174 ± 82	180 ± 84	168 ± 80	.156
Procedure time (min)	81 ± 54	91 ± 56	69 ± 49	<.001
Successful procedure (N)	422 (92%)	218(88%)	204(97%)	.001
RAK (Gy)	5.9 ± 4.6	6.1 ± 4.9	5.6 ± 4.2	.323
DAP (mGym^2^)	37.7 ± 29.2	38.6 ± 30.5	36.7 ± 27.7	.531
Fluoroscopy time (min)	57 ± 34	63 ± 35	50 ± 32	<.001
Stent size (mm)	2.8 ± 0.4	2.8 ± 0.4	2.8 ± 0.4	.315
Stent length (mm)	56 ± 28	60 ± 28	50 ± 27	.001

CTO, chronic total occlusion; DAP, dose area product; GW, guidewire; IVUS, intravascular ultrasound; LAD, left anterior descending; LCX, left circumflex; LM, left main; OCT, optical coherence tomography; PCI, percutaneous coronary intervention; RA, rotational atherectomy; RAK, reference air kerma; RCA, right coronary artery; SVG, saphenous venous graft.

An overall of three coronary perforations requiring pericardiocentesis, one limited aortic root dissection due to retrograde dissection from RCA which was medically stabilized without further operation, and one ventricular tachycardia requiring cardioversion possibly due to prolonged engagement of the guiding catheter occurred during the whole procedures.

### The success rate of intraplaque guidewire tracking for calcified versus noncalcified CTO according to the modified J-CTO score

**Figure 2.**
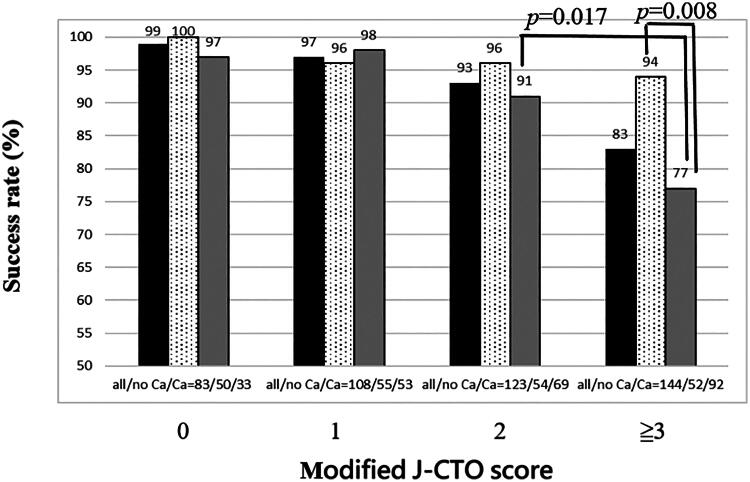
The success rate of intraplaque guidewire tracking for calcified versus non-calcified CTO lesions according to the modified J-CTO score. Ca, calcification; CTO, chronic total occlusion; ex., excluding.

For patients with noncalcified CTO and modified J-CTO scores of 0, 1, 2 and ≥3, the technique success rates were 100%, 96%, 96%, and 94%, respectively (Figure 2). For patients with calcified CTO lesions, the technique success rates were 97%, 98%, 91%, and 77% when the modified J-CTO scores were 0, 1, 2, and ≥3, respectively. Except for patients with calcified lesion and modified J-CTO score ≥3, the comparisons of success rate between those with each score and either the presence of calcification or not were statistically similar (all *p* > 0.05). The success rate only significantly declined in patients with calcified lesions when the modified J-CTO score was ≥3 (vs. score = 2, *p* = 0.017) and was significantly lower than that in patients with noncalcified CTO and a modified J-CTO score ≥3 (*p* = 0.008).

In patients with lesions of a modified J-CTO score ≥3, the presence of evident calcification was associated with a higher risk of procedural failure (OR = 5.1, 95% CI = 1.4–18.3, *p* = 0.012) after correcting for age, sex, prior coronary artery bypass graft, presence of CKD, all transradial access, and left circumflex artery in multivariate analysis (Table 3). Among all patients, the higher risk of procedural failure for calcified CTO lesions with a modified J-CTO score ≥3 also remained true (OR = 6.9, 95% CI = 3.4–14.1, *p* < 0.001) after correcting for age, sex, prior coronary artery bypass graft, and intrastent occlusion in multivariate analysis.

**Table 3.** Independent predictors of failed intraplaque GW tracking technique in patients with modified J-CTO score ≧3.

	Univariate	Multivariate	
	*P*-value	OR (95% CI)	*P*-value
Age	0.404	1.0 (0.98-1.06)	0.515
Male gender	0.422	2.0 (0.2-17.6)	0.537
CKD	0.063	0.24 (0.05-1.09)	0.064
CABG	0.044	3.6 (0.9-14.1)	0.061
All transradial access	0.029	0.39 (0.15-1.02)	0.056
LCX	0.085	0.28 (0.03-2.25)	0.228
Calcification	0.008	5.1 (1.4–18.3)	0.012

CABG, coronary artery bypass graft; CKD, chronic kidney disease; CTO, chronic total occlusion; GW, guidewire; LCX, left circumflex artery; OR, odds ratio.

### Comparisons of procedural time details in patients with successful intraplaque tracking and recanalization

**Figure 3.**
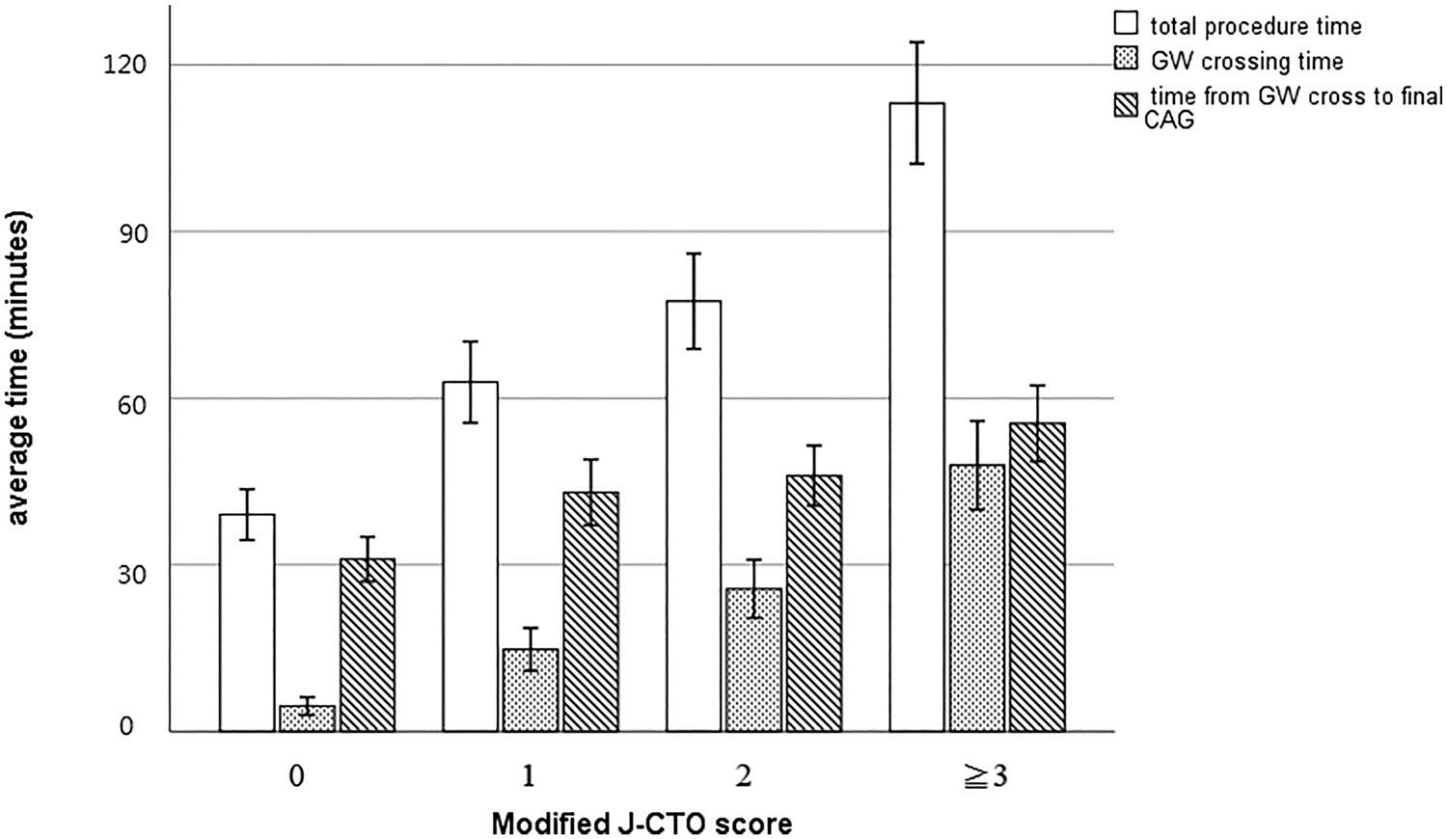
Comparisons of procedural time details in patients with successful intraplaque recanalization according to the modified J-CTO score. CAG, coronary angiogram; CTO, chronic total occlusion; GW, guidewire.

**Figure 4.**
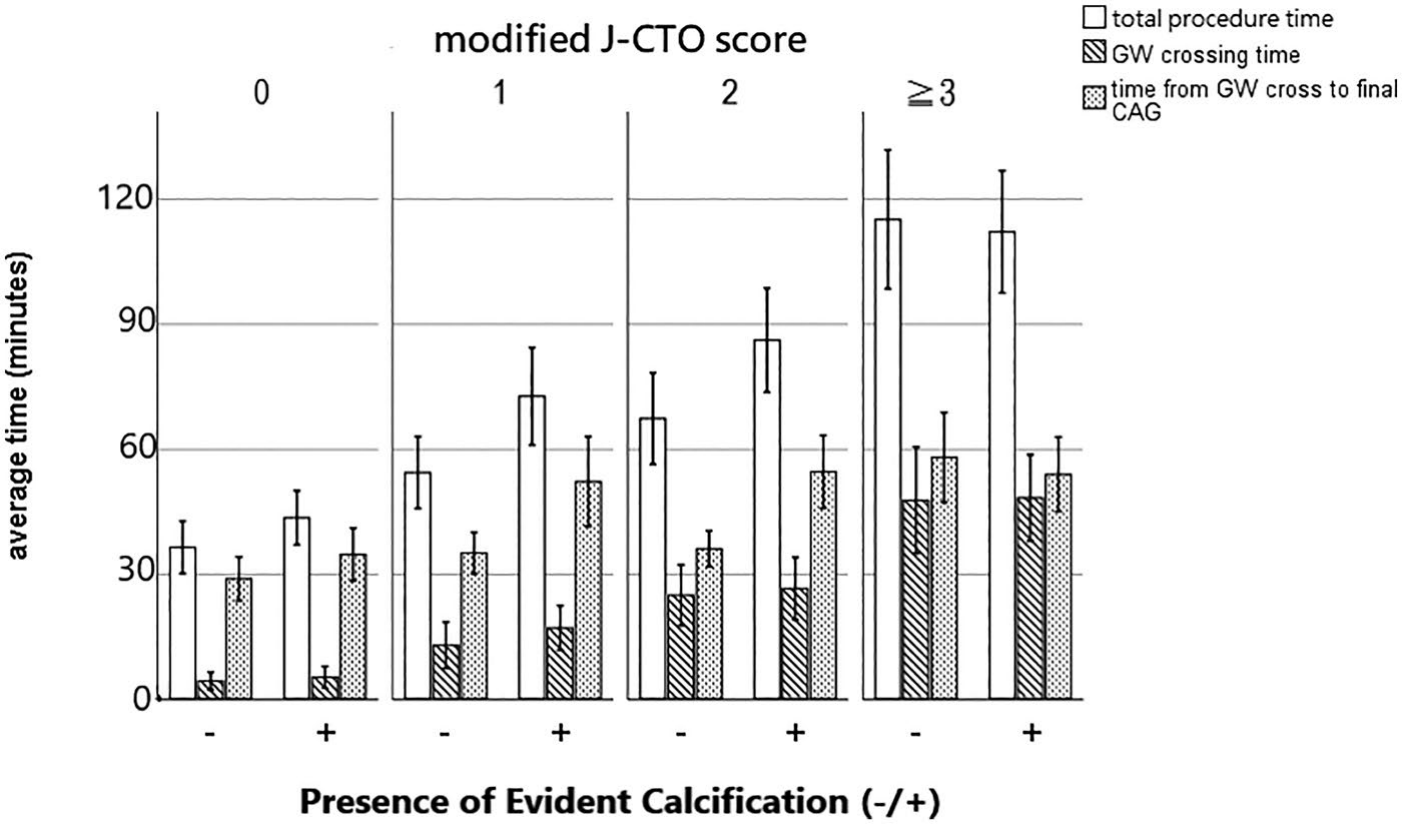
The procedural time comparisons for calcified versus non-calcified CTO lesions according to the modified J-CTO score. CAG, coronary angiogram; CTO, chronic total occlusion; GW, guidewire.

Among the 422 patients with successful procedures, the total procedure time, guidewire crossing time, and time from guidewire crossing to final coronary angiogram significantly increased as the modified J-CTO score increased (*p* < 0.001 for all comparisons by ANOVA) (Figure 3). For the J-CTO score, the probability of guidewire crossing time within 30 min was 98%, 94%, 76%, and 53% for score = 0, 1, 2, and ≥3, respectively, comparing with 98% (score = 0), 87% (score = 1), 71% (score = 2), and 43% (score ≥ 3), respectively for the modified J-CTO score. Areas under receiver-operator characteristic curves were comparable (J-CTO score: 0.77 versus modified J-CTO score: 0.78) between the J-CTO and modified J-CTO score. When the presence of calcification was on top of each modified J-CTO score (Figure 4), the total procedure time was longer in lesions with modified J-CTO scores 1 (72 ± 40 vs. 54 ± 31 min, *p* = 0.017) and 2 (86 ± 49 vs. 67 ± 39 min, *p* = 0.029), but was similar to those without calcification and modified J-CTO scores 0 (43 ± 18 vs. 36 ± 22, *p* = 0.133) and ≥3 (112 ± 60 vs. 118 ± 59 min, *p* = 0.572). As the guidewire crossing time was comparable between the calcified and noncalcified lesions with the same modified J-CTO score (score = 0: 5 ± 7 vs. 4 ± 7 min, score = 1: 17 ± 18 vs. 13 ± 20 min, score = 2: 26 ± 30 vs. 25 ± 26 min, score ≥ 3: 48 ± 42 vs. 48 ± 43 min, *p* > 0.1 for each comparison), prolonged procedure time associated with calcification in lesions with modified 1 and 2 was due to the longer time from guidewire crossing to final coronary angiogram (score = 1: 52 ± 37 vs. 35 ± 18 min, *p* = 0.003; score = 2: 54 ± 34 vs. 36 ± 15 min, *p* = 0.001).

In multivariate analysis, the presence of calcification independently predicted prolonged time from guidewire crossing to final angiography >60 min among lesions with modified J-CTO scores 1 and 2 (OR = 4.8, 95% CI = 2.2–10.2, *p* < 0.001) after correcting for age, sex, all transradial access, presence of blunt stump, ≥3 guidewires with different tip loads for antegrade wiring, and application of retrograde techniques (Table 4).

**Table 4.** Independent factors of prolonged time from GW cross to final CAG >60 min in patients with modified J-CTO score 1 and 2.

	Univariate	Multivariate	
	*P*-value	OR (95% CI)	*P*-value
Age	0.804	0.99 (0.96–1.02)	0.499
Male gender	0.861	1.2 (0.4–3.4)	0.756
All transradial	0.031	0.6 (0.3–1.3)	0.209
Calcification	<0.001	4.8 (2.2–10.2)	<0.001
Blunt stump	0.067	1.7 (0.9–3.4)	0.121
Antegrade GW≥ 3	0.019	5.5 (0.8–40.0)	0.090
Retrograde approach	0.044	8.3 (1.2–59.1)	0.034

CAG, coronary angiography; CTO, chronic total occlusion; GW, guidewire; OR, odds ratio.

## Discussion

Using intraplaque guidewire tracking techniques for CTO-PCI, this study demonstrated that a much-declined rate of procedural success caused by calcification occurred mainly in lesions with the highest difficulty. However, calcification was only related to prolongation of angioplasty/stenting time in lesions with moderate difficulty. The comparable guidewire crossing time despite the presence of calcification suggested the potential facilitation of calcification as the landmark for guidewire tracking. Since most CTO interventionists usually have their own manner of recanalization techniques, these findings suggest that the interpretation of calcification during CTO-PCI should be more cautious because of its technique-dependent character.

Several CTO scoring systems [6, 8–11] included calcification as a fundamental factor in predicting angiographic success. In contrast, the Prospective Global Registry for the Study of Chronic Total Occlusion Intervention (PROGRESS) CTO score [19] does not suggest that calcification is a predictor of technical failure. Among several possible causes leading to diverse contribution of calcification on predicting the feasibility of CTO interventions, the percentage of “dissection and reentry” devices and techniques used should be a key factor. The presence of calcification suggests a longer duration and harder tissue composition in occluded lesions [3]. The application of “dissection and reentry” methods, which potentially bypass the calcified or most resistant segment with either devices or reverse CART techniques, can be less relevant to the presence of calcification. For example, although the technical details were not mentioned, the J-CTO score registry included 25.7% of retrograde procedures,^8^ compared with 30.2% of antegrade dissection/reentry and 37.4% of retrograde procedures in the PROGRESS-CTO registry [19]. Thus, the percentage of “dissection and reentry” methods involved in CTO interventions would partially compromise the true effect of calcification on the feasibility of recanalization.

On the contrary, the presence of calcification was theoretically thought to be more challenging to intraplaque guidewire tracking techniques. However, experienced interventionists usually depended upon the guidance by the distribution of calcification within the occluded segment for guidewire tracking. In the study, we showed a comparable success rate of lesions with modified J-CTO score < 3, irrespective of the presence of calcification. Although the calcified occlusion was considered harder, a substantial amount of loose tissue remained surrounding the calcification along the entire occluded segment. Additionally, linear calcification or calcium at multiple spots could facilitate the guidewires in tracking the relatively soft tissue beside the calcification through the assumed course of the CTO. With these wiring techniques, the disadvantage of calcification is partially attenuated, as demonstrated in the present study. Nevertheless, we still showed a significantly decreased rate of recanalization success to <80% in lesions with calcification and a modified J-CTO score ≥3. Among lesions with a modified J-CTO score ≥3, the presence of calcification was shown to be independently associated with a 5.1-fold risk of intraplaque tracking and recanalization failure after multivariate corrections. Among the whole cases, the overall percentage of retried lesions was 14%, far less than that of approximately 50% with respect to the other three parameters of the modified J-CTO score. This suggests that the disadvantageous effect of calcification on recanalization feasibility would be more conspicuous for the highest complex occlusions characterized by simultaneous existence of blunt entry, bending >45°, and length ≥2 cm as the majority. Due to the significantly decreased success rate with intraplaque guidewire tracking techniques for more complex lesions with calcium shown in the current study, an earlier switch to other revascularization strategies should be considered for such kind of lesions.

The presence of calcification has been shown to be associated with longer procedure time for CTO interventions [5]. We did further analysis of the two major time components of the recanalization procedures, including the guidewire crossing time and angioplasty/stenting time (time from guidewire crossing to final angiogram). We revealed a comparable guidewire crossing time between noncalcified and calcified occlusions with each modified J-CTO score. If the calcification point was added to the modified J-CTO score, the real J-CTO score was higher in each subgroup with calcified CTO than in those without calcified CTO. The lack of prolongation of guidewire crossing, even with a higher J-CTO score due to the presence of calcification, again emphasized the potential benefit of calcification as a landmark during intraplaque tracking. This further suggested that the interpretation of J-CTO score ≥2, traditionally serving as the cut-off point for the feasibility of guidewire crossing within 30 min, should be cautious when calcification was present and guidewire tracking techniques were performed.

In the current study, there was a 4.8-fold risk of angioplasty/stenting time >60 min when treating calcified occlusions with modified J-CTO scores 1 and 2 in multivariate analyses. Calcified coronary lesions usually require more dedicated lesion preparation with extension catheters, smaller or noncompliant balloons, cutting balloons, and even rotation atherectomy before stenting, leading to more procedural time after guidewire crossing. For example, among 122 calcified occlusions with modified J-CTO scores 1 and 2, rotational atherectomy was performed for 11 (9%) lesions, which would partly contribute to the time prolongation [20]. For occlusions with modified J-CTO ≥3, although rotational atherectomy was performed for 4 of the 71 (5.6%) calcified lesions, retrograde techniques were also performed in 11 of the 49 (22%) noncalcified lesions compared with 13% (9/71) of calcified lesions. When the retrograde approach was used for recanalization, several steps including manipulating the retrograde guidewire into the antegrade guiding catheter, advancing the microcatheter through the occluded lesion and into the antegrade guiding catheter, and then performing the guidewire externalization were all necessary. However, difficulties were sometimes and possibly encountered during these steps and would lead to more time-consuming, leading to the comparable angioplasty/stenting time between noncalcified and calcified lesions with a modified J-CTO ≥3.

### Study limitations

This study has some limitations. First, since CTO-PCI is a highly operator- and technique-dependent procedure, the study results could be altered accordingly. Nonetheless, this study aimed to investigate the role of calcification in the J-CTO score using a basic wiring-based CTO recanalization strategy to minimize the potential heterogeneity in registries. Second, the judgment of intraplaque tracking and crossing was partly based on the guidewire tactile feedback of the operators because the application of intravascular imaging was 45%. Basically, the judgment made by an experienced operator usually could be relatively reliable, particularly for shorter or simpler occlusions with a lower J-CTO score. One study suggested the discordance between presumed and IVUS-confirmed true lumen was 15.8% [21]. Furthermore, since preservation of the sizable side-branches within the occluded segment would be essential to CTO recanalization and the key purpose of intraplaque tracking, the definition of procedural success, including the patency of side branches ≥1.5 mm from 5 mm before to 5 mm after the occluded segment, would further suggest limited subintima creation, if any, and possibly ensure the high likelihood of intraplaque tracking in the study. Third, we did not classify all lesion into four group with non, mild, moderate, or severe calcification due to the concern of patient number enrolled, and the impact of calcification severity on the results could not be fully answered. Nevertheless, our study was still comparable to most CTO-PCI studies [7–9], which usually defined calcification as none/mild and moderate/severe. Forth, the difficulty of intraplaque tracking with the presence of calcification by retrograde guidewire manipulation must differ from that by antegrade wiring. Whether the study results would change when a higher percentage of retrograde techniques is included for analyses cannot be answered and may deserve further investigation. Nevertheless, the lower rate of retrograde techniques in the study would at least minimize the different effect between antegrade and retrograde wiring, and the study result was still applicable to the real-world practice since the antegrade wiring techniques remain to be relatively accessible, safer, and applied more for CTO recanalization.

## Conclusions

This study suggests that when wiring-based intraplaque tracking techniques are applied to CTO interventions, more cautious interpretation of calcification with respect to recanalization feasibility or procedure time is deserved due to its potential technique-dependent character. If the impacts of the other parameters of the J-CTO score would be altered with different recanalization techniques applied to CTO interventions deserve further investigations.

## Data Availability

The data that support the findings of this study are available from the corresponding author upon reasonable request.
